# No net effect of host density on tick‐borne disease hazard due to opposing roles of vector amplification and pathogen dilution

**DOI:** 10.1002/ece3.9253

**Published:** 2022-09-06

**Authors:** Sara Gandy, Elizabeth Kilbride, Roman Biek, Caroline Millins, Lucy Gilbert

**Affiliations:** ^1^ Institute of Biodiversity, Animal Health and Comparative Medicine University of Glasgow Glasgow UK; ^2^ Institute of Infection, Veterinary and Ecological Sciences University of Liverpool Liverpool UK

**Keywords:** *Borrelia burgdorferi*, dilution effect, host community, Lyme disease, ticks, transmission hosts

## Abstract

To better understand vector‐borne disease dynamics, knowledge of the ecological interactions between animal hosts, vectors, and pathogens is needed. The effects of hosts on disease hazard depends on their role in driving vector abundance and their ability to transmit pathogens. Theoretically, a host that cannot transmit a pathogen could dilute pathogen prevalence but increase disease hazard if it increases vector population size. In the case of Lyme disease, caused by *Borrelia burgdorferi* s.l. and vectored by Ixodid ticks, deer may have dual opposing effects on vectors and pathogen: deer drive tick population densities but do not transmit *B. burgdorferi* s.l. and could thus decrease or increase disease hazard. We aimed to test for the role of deer in shaping Lyme disease hazard by using a wide range of deer densities while taking transmission host abundance into account. We predicted that deer increase nymphal tick abundance while reducing pathogen prevalence. The resulting impact of deer on disease hazard will depend on the relative strengths of these opposing effects. We conducted a cross‐sectional survey across 24 woodlands in Scotland between 2017 and 2019, estimating host (deer, rodents) abundance, questing *Ixodes ricinus* nymph density, and *B. burgdorferi* s.l. prevalence at each site. As predicted, deer density was positively associated with nymph density and negatively with nymphal infection prevalence. Overall, these two opposite effects canceled each other out: Lyme disease hazard did not vary with increasing deer density. This demonstrates that, across a wide range of deer and rodent densities, the role of deer in amplifying tick densities cancels their effect of reducing pathogen prevalence. We demonstrate how noncompetent host density has little effect on disease hazard even though they reduce pathogen prevalence, because of their role in increasing vector populations. These results have implications for informing disease mitigation strategies, especially through host management.

## Graphical abstract

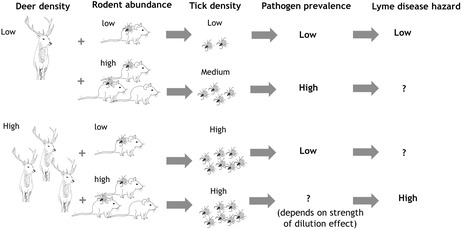

## 1 INTRODUCTION

Vector‐borne diseases, which account for 22% of emerging infectious diseases (Jones et al., 2008), rely upon vectors to carry and transmit pathogens from one host to another. When vectors feed on multiple host species, the disease ecology system can be highly complex with different hosts playing different roles in feeding vectors and transmitting pathogens. There may also be ecological interactions between hosts in an ecosystem, with one host species affecting the abundance of another through competition or predation (Hoyer et al., 2017; Levi et al., 2012). It can therefore be extremely challenging to tease apart which hosts and which interactions are most important in determining disease risk. One intriguing aspect of these complex ecological interactions is that they could theoretically result in a seemingly contradictory situation where a host that cannot transmit the pathogen can increase the environmental hazard of disease (Gandy et al., 2021).

Disease hazard is defined as the density of infected vectors, the product of vector density, and pathogen prevalence (Kilpatrick et al., 2017). Thus, the abundance of suitable vector hosts can determine how many vectors successfully complete their life cycle and many studies have shown that host density can drive vector populations (Gilbert et al., 2012; Mysterud et al., 2016; Pacilly et al., 2014). On the other hand, pathogen prevalence (the proportion of vectors that are infected) is influenced by the proportion of immature vectors that feed on infected transmission hosts (van Duijvendijk et al., 2017; Vuong et al., 2017). Pathogen prevalence is likely to be influenced by the density of hosts that maintain and transmit the pathogen relative to hosts that cannot transmit the pathogen. Vectors feeding on such “non‐competent” hosts will not become infected and it has been hypothesized that an increase in noncompetent hosts is one mechanism that could reduce (or “dilute”) pathogen prevalence by diverting vectors away from feeding on pathogen transmission hosts (Norman et al., 1999; Ostfeld & Keesing, 2000a, 2000b).

Environmental disease hazard is thus expected to depend on a combination of vector reproduction host, pathogen transmission host, and noncompetent host abundance. Of particular interest to disease ecology theory is that, when an animal acts as both a vector reproduction host and a noncompetent pathogen host, it could simultaneously increase vector populations while decreasing (diluting) pathogen prevalence (Gandy et al., 2021; Gilbert et al., 2001). The relative strengths of these opposing effects will largely determine the resulting environmental disease hazard, creating an intriguing ecological scenario whereby noncompetent hosts could, theoretically, increase disease hazard through amplifying vector density. However, the complexity of the ecological interactions between pathogens, vectors, and the host community make it challenging to predict under what conditions this might be the case.

Lyme disease, the most prevalent vector‐borne disease in the Northern hemisphere (Steere et al., 2007), is an Ixodid tick‐borne zoonosis caused by bacteria belonging to the group *Borrelia burgdorferi* sensu lato (Steere et al., 2007). It is an ideal system to test hypotheses about complex host‐vector‐pathogen ecology because the Ixodid tick vector feeds on a wide range of animal host species. In Europe, pathogen transmission is primarily through *Ixodes ricinus* ticks (Piesman & Gern, 2004), which have three life‐stages (larva, nymph, and adult). Larvae and nymphs feed on most terrestrial vertebrate species, while adult females usually take a blood meal from large mammals before laying their eggs. Rodents are the most important hosts for immature ticks and can feed up to 89% of larvae (Hofmeester et al., 2016; Tälleklint & Jaenson, 1994) followed by birds, feeding up to 5% of larvae (Hofmeester et al., 2016). Deer are an important source of blood meals for both immature and adult female ticks and often drive tick densities (Deblinger et al., 1993; Gilbert et al., 2012; Kilpatrick et al., 2014; Ruiz‐Fons & Gilbert, 2010). In Europe, it was suggested that roe deer can contribute to feed up to 3% of larvae and 15% of nymphs (Tälleklint & Jaenson, 1994), while two other studies that inspected the entire body of roe deer found, on average 11 larvae and 24 nymphs per animal in Germany and 11 larvae and 31 nymphs per animal in Spain (Kiffner et al., 2011; Vasquez et al., 2011). Thus, these results suggest that large ungulate could be important source of blood meals for immature ticks (Gandy et al., 2021).

Several host types can transmit *B. burgdorferi* s.l. in the United Kingdom (UK), each maintaining different genospecies of the pathogen. Rodents can transmit *B. afzelii* (Hanincová, Etti, et al., 2003) while many bird species can transmit *B. garinii* and *B. valaisiana* (Hanincová, Taragelová, et al., 2003). *Borrelia burgdorferi* s.s. is associated with a variety of hosts (Kurtenbach et al., 1998), including squirrels (*Sciurus* spp.) (Millins et al., 2015). While rodents, birds, and squirrels are the main transmission hosts for the various genospecies of *B. burgdorferi* s.l. in Europe, roe (*Capreolus capreolus)* and red deer (*Cervus elaphus*) are unable to transmit *B. burgdorferi* s.l. (Jaenson & Tälleklint, 1992; Kurtenbach et al., 2002). Deer, therefore, are particularly interesting hosts for Lyme disease ecology as they could potentially play opposing roles in shaping Lyme disease hazard (the density of infected nymphs), by increasing vector densities while not transmitting *B. burgdorferi* s.l. The relationship between deer density and Lyme disease hazard is likely to be shaped by the abundance of nontransmission hosts as well as deer densities, which will vary widely across environments.

**FIGURE 1:**
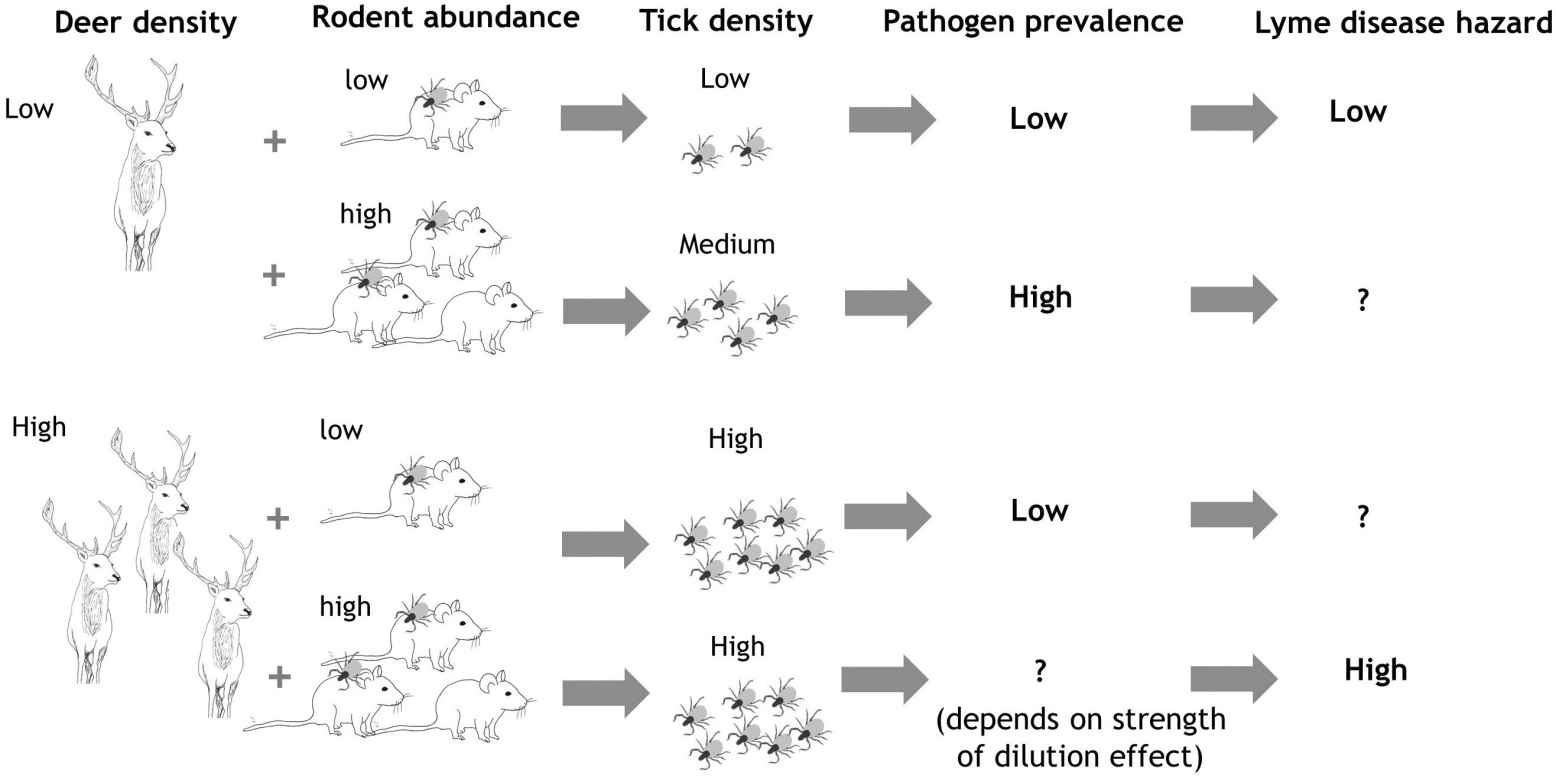
Conceptual diagram illustrating the predicted effects of deer and rodent densities on the density of nymphs, nymphal infection prevalence, and Lyme disease hazard

The aim of this study is to test the effects of deer on Lyme disease hazard over a wide range of deer densities while simultaneously accounting for varying rodent densities, in order to gain mechanistic insight through the effects of deer and rodent abundance on tick density and pathogen prevalence. This study focused on rodents as the main transmission hosts because they feed a large proportion of larvae and because *B. afzelii* is the most abundant genospecies causing human disease in Europe (James et al., 2013; Mannelli et al., 2012; Michelet et al., 2014; Millins et al., 2016). Here, we investigate how host abundance affects nymph density and pathogen prevalence, at first separately and then combined as disease hazard. We predict (i) a positive correlation between nymph density and host abundance (deer and rodent) and (ii) that nymphal infection prevalence for *B. burgdorferi* s.l. should decrease with increasing deer densities due to a dilution effect. We expect this effect as a higher proportion of larvae should feed on deer when they are present at high density. Regarding Lyme disease hazard, we predict that high deer densities could either (iii) increase disease hazard, if their role of increasing the density of nymphs is stronger than that of diluting pathogen prevalence, or (iv) reduce disease hazard, if their role of diluting pathogen prevalence is stronger than that of increasing the density of nymphs (Figure 1). We predict that (v) both infection prevalence and disease hazard for *B. afzelii* will be higher in sites with high rodent abundance (Figure 1).

## 2 MATERIALS AND METHODS

### 2.1 Study sites

**FIGURE 2:**
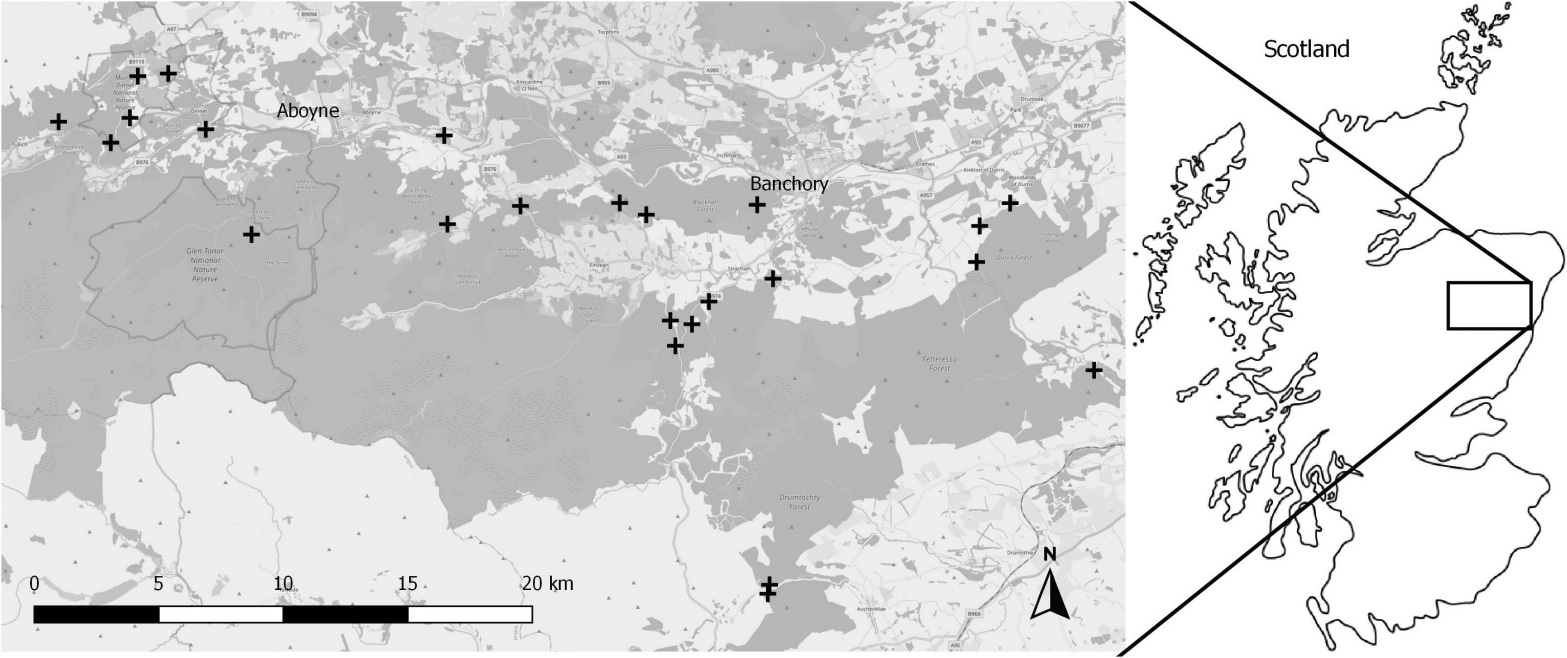
Map showing the 24 sites surveyed for this study located in Northeast Scotland

We used 24 sites located in Aberdeenshire, Northeast Scotland (central point: 57.000°N, 2.700°W, Figure 2) selected specifically to cover as wide a range of deer densities as possible. Fifteen sites were surveyed in 2017 and 2018 and nine sites were surveyed in 2018 and 2019. To minimize any potential effects of microclimatic differences on tick density, pathogen prevalence, and Lyme disease hazard (Gilbert, 2010), woodland sites (17 coniferous, 3 deciduous, and 4 mixed) were selected within a narrow altitudinal range (112–274 m), were at least 1 km apart and within 50 km of each other (Table S1). We used a one‐year time‐lag between host density estimation and tick collection as questing nymphs collected at year_t_ will have fed on a host (and acquired any infection) as larvae at year_*t*−1._

### 2.2 Estimation of deer density

An index for deer density was estimated the first year of data collection (2017 for fifteen sites and 2018 for nine sites) using the standing crop plot count method (Mayle et al., 1999). Within each site, twenty 10 m × 1 m transects, separated from one another by at least 20 m, were randomly generated using QGis software (QGIS Development Team, 2016). In May, the number of red and roe deer pellet groups were counted along each transect. An index for deer density was calculated for each site using our dung counts and published defecation and decay rates (Mandujano, 2014). Defecation rates for red deer were taken as 24.88 per day, (Mitchell, 1984), and for roe deer as 19.33 per day (Mitchell et al., 1985). We used a minimum pellet decay period of 189 days and maximum of 416 days (Mayle et al., 1999). To validate this method, we applied it to pellet group counts from a local deer farm with a known stocking density of 32.47 individuals per km^2^; our estimate was 30.81 deer per km^2^, confirming lack of bias.

### 2.3 Estimation of rodent abundance

Rodent abundance was estimated at each site during the first year of data collection (2017 for 15 sites and 2018 for nine sites) and the two woodland species present in Scotland were targeted; wood mice (*Apodemus sylvaticus*) and bank voles (*Myodes glareolus*). Because different methods of estimating rodent abundance can be biased by ground vegetation type (e.g., we found rodent signs were much easier to detect in grass, whereas live trapping success was much lower in grass than in other ground vegetation types), we used two methods.

First, rodent abundance (number of rodents per 100 trap nights) was quantified at each site by live‐trapping (Bouchard et al., 2013). To minimize the effect of seasonal variations in rodent populations, trapping was done at all study sites within 40 days in June and July. Ten to fifteen 100 ‐m long transects separated by 10 m were set up at each site in a grid pattern. Ten nonselective Sherman traps (16 × 5 × 6.5 cm HB Sherman Inc., Tallahassee, Flor.) were installed every 10 m on each trapping line and baited with oats for two nights; therefore, there were 200–300 trap nights per site. Shrews (*Sorex* spp.) are known to harbor *B. afzelii* and feed larval ticks (Brisson & Dykhuizen, 2004) and thus, we would have liked to estimate their abundance. However, due to UK trapping regulations, we could not capture shrews and traps were equipped with small holes, allowing them to escape. Traps were activated after 17:00 h and checked every morning before 10:00 h. Species, sex, weight, and age category (juvenile or adult) of each individual rodent were recorded and ticks attached were counted. All trapped individuals were released at the capture site. We calculated the number of larvae fed by combining larval burden by the relative abundance of rodents.

The second method used to assess rodent abundance was by recording vole signs (tunnels and holes) at each site in May on the first year of data collection on the 10 m × 1 m transects used for the deer density estimation. The average number of vole signs observed per 10 m^2^ for each site was used for analysis.

We then created a simple index of rodent abundance which combined data from both methods. First, we classified each site as having either low or high rodent abundance from each method. The data from each method exhibited a bimodal distribution with a clear gap between low and high density (Figure S1). If a site scored a high category for at least one index, it was defined as a high rodent abundance site, whereas sites which scored low categories for both indexes were identified as low rodent abundance (Table S1). As it has been suggested that bank voles and wood mice do not differ in their competence for transmitting *B. afzelii*, we grouped both species into one rodent abundance index (Kurtenbach et al., 1998).

### 2.4 Questing *Ixodes ricinus* nymph surveys

Questing nymphs were collected three times a year (May, July, and September) for each year following host density estimation (2018 and 2019) using a standard blanket dragging method (Falco & Fish, 1992). A white 1 m × 1 m square of fleece blanket material was dragged over vegetation along 10 m long transects. At each site, twenty transects were randomly surveyed and separated from each other by at least 20 m. Nymph ticks on the blanket were counted, collected, and kept at −20°C for pathogen analysis. After carrying out the 20 transects, dragging was continued, if needed, until at least 100 nymphs were collected at each site visit or for a maximum of 3 h of additional dragging. This was to ensure enough nymphs were collected for robust pathogen prevalence estimate; we used the formula developed by Daniels (Daniels, 1999), based on an average prevalence of 1.7–5.6% (James et al., 2013; Millins, 2016) to calculate the sample size needed. Woodland type (coniferous, deciduous, or mixed woodland) was recorded at the site level while ground vegetation was classified into four categories: (1) grasses and herbaceous species, (2) *Ericaceous* species (*Calluna* and *Vaccinium*), (3) moss species, and (4) bracken and ferns (Millins, 2016). The dominant vegetation category (that with the most cover) over each transect was recorded. Vegetation height was measured at the beginning (1 m), middle (5 m), and end (10 m) of each transect, and the mean for each transect was included as a continuous variable in analysis of tick density as it can affect dragging efficiency (Gilbert, 2010). Tick surveys were conducted between 0900 hours and 1800 hours and air temperature, relative humidity and time were also recorded for each transect, as these may affect the proportion of nymphs questing. For analysis, we also used the rainfall recorded the day before ticks were collected from the nearest weather station at Aberdeen Airport (http://rp5.co.uk/Weather_archive_in_Aberdeen_[airport]_UK).

### 2.5 Estimation of *B. burgdorferi* s.l. prevalence

Nymphs were extracted individually using an ammonia extraction method (Gern et al., 2010). *Borrelia burgdorferi* s.l. was detected from samples using a qPCR protocol on fragments of OspA genes based on the protocol described by Heylen et al. (2013). The protocol was optimized using the IQ™ Supermix (Bio‐Rad Laboratories, Hercules, USA) in a Stratagene Mx3005P thermal cycler (Agilent, Santa Clara, US). Each reaction contained IQ™ Supermix, two primers at 200 nM (B‐OspA_modF: AATATTTATTGGGAATAGGTCTAA and B‐OspA_borAS: CTTTGTCTTTTTCTTTRCTTACAAG), the probe (B‐OspA_mod: FAM‐AAGCAAAATGTTAGCAGCCTTGA‐BHQ‐1™) at 100 nM and 3 μl of DNA. One positive and one negative control were added for every 94 samples. To identify the genospecies, samples which tested positive were then tested using a nested PCR protocol targeting the 5S‐23S intergenic spacer region (Rijpkema et al., 1995). Each positive sample was separated from another by a negative control and samples were visualized on 2% agarose gel containing ethidium bromide in Tris‐borate EDTA buffer. Positive samples were sent to Edinburgh genomics for Sanger sequencing to identify the genospecies of *B. burgdorferi* s.l. present.

### 2.6 Statistical analyses

All statistical analyses were performed in the software R version 3.5.1 (R Core Team, 2013) using the glmmTMB (Brooks et al., 2017), lme4 (Bates et al., 2015), and MuMIn (Barton, 2019) packages.

#### 2.6.1 Statistical modeling

For Generalized Linear Mixed Effects Models (GLMMs), we assessed potential collinearity between explanatory variables using variance inflation factors (VIFs) (Zuur et al., 2009) and we removed variables with a VIF above 4. For response variables that were counts (tick numbers), we fitted GLMMs with either a Poisson or negative binomial distribution and we checked whether a zero‐inflation model was better suited using the zero‐inflation function from the DHARMa package (Hartig, 2020). Model selection was done using the dredge function from the MuMIn package based on the corrected Akaike Information Criterion (AICc) (Brewer et al., 2016). When a significant fixed effect was a multi‐level categorical variable, we conducted post hoc Tukey tests to identify which pairwise comparisons were different from each other.

#### 2.6.2 The effect of deer and rodent densities on questing nymph density

To investigate the effect of deer density on questing nymph density, we used a GLMM with a Poisson distribution and the number of nymphs per transect as our response variable. The full model included rodent abundance (high or low) the previous year, deer density the previous year (red and roe deer combined), month (May, July, September), ground vegetation type (grasses, *Ericaceous* spp., mosses, ferns), woodland type (deciduous, coniferous, mixed forest), rainfall the previous day and temperature in its quadratic form (as both can affect the proportion of ticks questing), ground vegetation height in its quadratic form and whether the ground was wet during tick collection (as they affect dragging efficiency). We also added the interaction between deer density the previous year and woodland type as woodland type might affect how deer use their environment and thus, their effect on nymph density. Roe and red deer were combined as one variable because both species play a similar role as vector reproduction hosts while not transmitting *B. burgdorferi* s.l. (Pacilly et al., 2014). Site was added as a random effect and an observation level random effect was included to account for overdispersion (Elston et al., 2001; Harrison, 2014).

#### 2.6.3 The effects of deer and rodents on nymphal infection prevalence

To test for the effect of deer and rodent abundance on pathogen prevalence, we built two GLMMs: one to test the effect of deer on the complex of Lyme disease pathogens (*B. burgdorferi* s.l.) and one to test the effect of both deer and rodents on the rodent‐associated Lyme disease pathogen (*B. afzelii*). For both GLMMs we specified a binomial distribution, and both analyzed at the site visit level (three visits per site). For the first model, *B. burgdorferi* s.l. prevalence (number of infected nymphs over the number of uninfected nymphs) was used as the response variable and the full model included deer density the previous year, woodland type, month, and the interaction between deer density and woodland type.

In the second model, the response variable was the *B. afzelii* prevalence (number of infected nymphs over the number of uninfected nymphs) and the full model included rodent abundance (high or low) the previous year, deer density the previous year, month, and woodland type. As described above, we included the interaction between deer and woodland type and the interaction between deer density and rodent abundance to examine how deer and rodent densities might interact to shape pathogen prevalence. For both models, site was added as a random effect and an observation level random effect was added to account for overdispersion (Harrison, 2015).

#### 2.6.4 The effects of deer and rodents on Lyme disease hazard

Similarly, two models (one for *B. burgdorferi* s.l. and one for *B. afzelii*) were used to assess the effects of deer and rodents on Lyme disease hazard. The first model focused on the effect of deer on *B. burgdorferi* s.l. and we used a zero inflated GLMM with a negative binomial distribution. The response variable was the density of infected nymphs (three estimates per site) and we used an offset for the area surveyed (Zuur et al., 2009). The full model included deer density the previous year, month, woodland type, ground vegetation height, rainfall the previous day and temperature (as both can affect the proportion of ticks questing), and an interaction between deer density and woodland type. For the second model that simultaneously examined the effects of rodents and deer together, we used the density of infected nymphs with *B. afzelii* as our response variable, with an offset accounting for the area surveyed. The full model included deer density the previous year, rodent abundance the previous year, month, woodland type, ground vegetation height, temperature, rainfall the previous day, the interaction between deer density and rodent abundance and the interaction between deer density and woodland type. For both models, site was added as a random effect.

## 3 RESULTS

**FIGURE 3:**
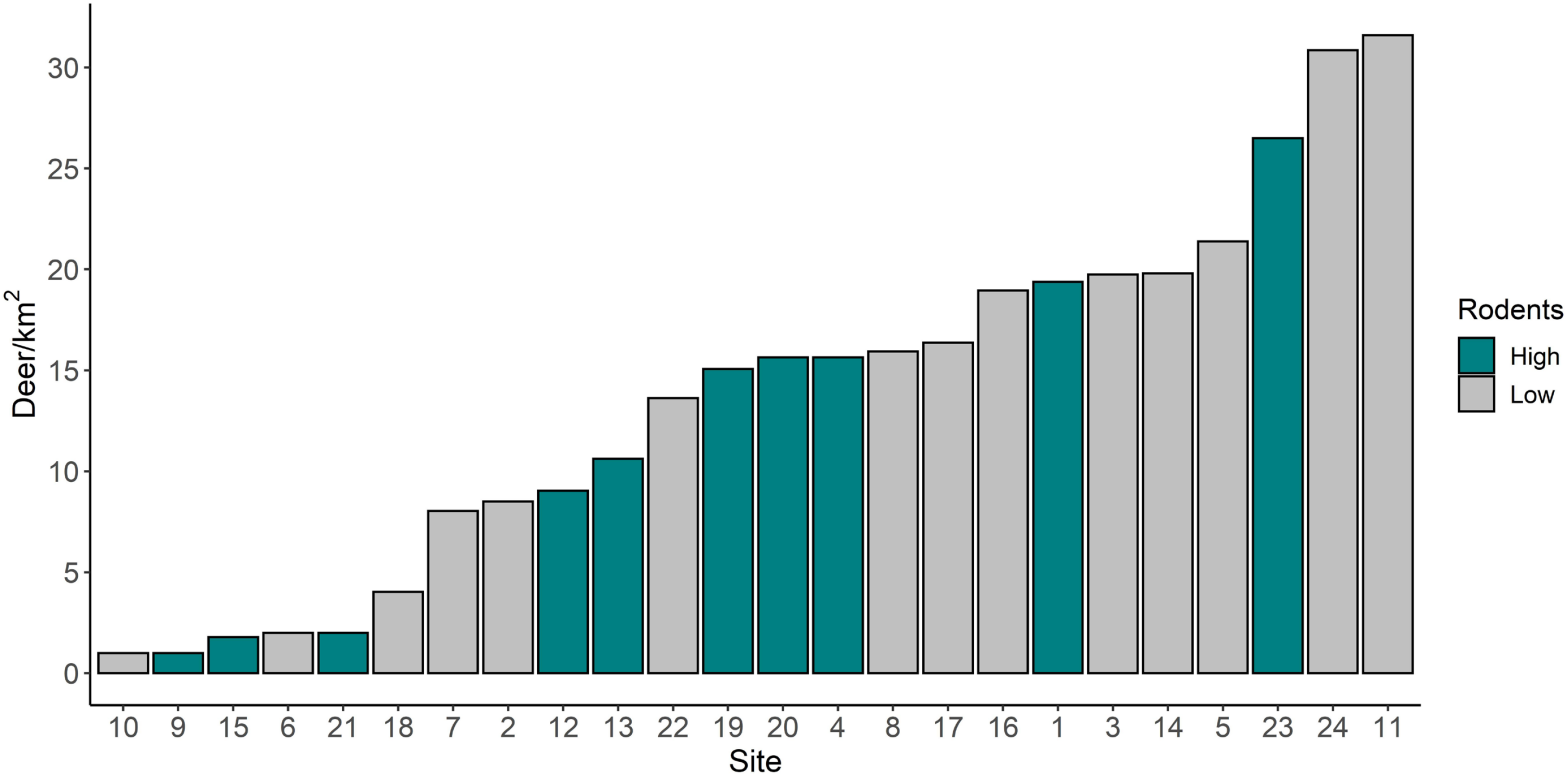
Deer per km^2^ at each of the 24 sites. Rodent abundance category (green = high; grey = low) is also shown

The estimated density of deer (red and roe combined) ranged from 1 to 31.6 deer/km^2^ (mean: 13.7, SD: 8.9) (see Figure 3 and see Table S1 for deer density in each site). In total, 54 bank voles (26 females, 24 males, and 4 undetermined) and 47 wood mice (15 females and 32 males) were captured over 5166 trap nights. On average, 2.02 (range: 0–13.6, SD: 3.1) rodents were captured per 100 trap nights. We calculated the number of larvae fed by rodents (larval burden x relative abundance of rodents using trapping data) and rodents fed 24.4 larvae (SD: 50.5). Sites with high rodent abundance fed 33.4 larvae (SD: 61.4) compared to 9.1 larvae (SD: 13.5) in sites with low rodent abundance (Figure S2). A total of 5230 questing nymphs were counted from 1438 transects and, on average, 36 nymphs were counted per 100 m^2^ (range: 0–410, SD: 41). A random subset of 2500 nymphs was examined under the microscope for species identification using specific keys (Márquez et al., 1992) and they were all identified as *Ixodes ricinus*. Thus, it was assumed that all the ticks collected from blanket dragging were *I. ricinus*.

### 3.1 The effects of deer and rodent densities on questing nymphs

The selected model investigating the effects of deer density and rodent abundance index on questing nymph density included deer density the previous year, rodent abundance the previous year, woodland type, ground vegetation type, month, whether the ground was wet, rainfall the previous day, ground vegetation height, and temperature (Table 1).

**TABLE 1:** Outputs from the generalized linear mixed effect model explaining the effects of deer, rodents, and environmental factors on the density of questing *Ixodes ricinus* nymphs

	Estimate (log)	SE	*z*‐value	*p*‐value	ΔAICc^a^
Intercept	0.81	0.21	3.78	<.001	
Deer density year_*t*−1_	0.45	0.10	4.45	<.001	12.6
Rodent abundance year_*t*−1_: low (baseline: high)	0.49	0.19	2.54	.01	3.7
Ground vegetation (baseline: Bracken/ferns)					
Ericaceous shrubs	−0.06	0.15	−0.37	.71	15.5
Grasses	−0.41	0.15	−2.78	.005	
Mosses	−0.55	0.22	−2.51	.01	
Woodland type (baseline: coniferous)					
Deciduous	0.58	0.30	1.91	.06	1.9
Mixed	−0.42	0.29	−1.46	.14	
Ground Vegetation height	−0.09	0.05	−1.72	.07	1.0
Ground Vegetation height^2^	−0.03	0.02	−1.83	.06	1.4
Month (baseline: July)					
May	0.14	0.07	1.88	.06	17.8
September	0.33	0.07	4.75	<.001	
Temperature	−0.17	0.04	−4.11	<.001	14.3
Temperature^2^	−0.03	0.02	−1.68	.10	0.7
Ground wet: yes (baseline: not wet)	−1.09	0.11	−9.81	<.001	93.8
Rainfall (mm) previous day	−0.07	0.03	−2.59	.01	4.5

^a^ The ΔAICc refers to the effect of removing the variable in the given row on the AICc of the best model. For example, a ΔAICc of 10 means that the AICc of the model increased by 10 after removing the variable.

**FIGURE 4:**
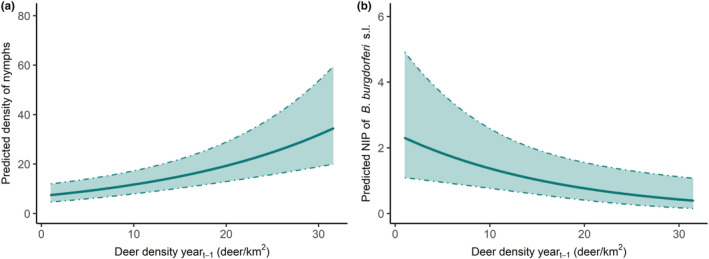
(a) Predicted density of questing nymphs (per 100 m^2^) depending on deer density the previous year (ΔAICc of 12.6 if deer is removed from the selected model) and (b) predicted nymphal infection prevalence for *Borrelia burgdorferi* s.l. (%) depending on deer density the previous year (ΔAICc of 4.1 if deer is removed). Shaded bands represent 95%CI

There was a significant positive correlation between deer density and questing nymph density (*p* < .001), for which nymph density increased by 1.0/100 m^2^ for every unit increase in deer density (individuals/km^2^) (Figure 4a). Questing nymph density was higher in sites with a low rodent abundance (predicted density: 35.2/100 m^2^, 95%CI: 19.1–66.3) index compared to sites with a high rodent abundance index (predicted density: 21.5/100 m^2^, 95%CI: 11.3–41.7, *p* = .01).

Questing nymph density was also influenced by other variables: more nymphs were questing in September compared to May (*p* = .02) and July (*p* < .001) and more nymphs were counted on blanket drag transects if the ground was dry (*p* < .001) (see Table S2 for effect sizes and Table S3 for Tukey‐tests results). Nymph density in deciduous woodlands was higher than in mixed woodlands (*p* = .03) but similar to coniferous forests (*p* = .13) (Tables S2, S3). Regarding dominant ground vegetation, questing tick density in *Ericaceous* species was higher than in grasses (*p* < .001) or mosses (*p* = .02) but similar in ferns (*p* = .98) (Tables S2, S3). Nymph density was similar between moss and grass (*p* = .80). Finally, more questing nymphs were counted with cooler temperatures (*p* < .001), lower vegetation height (*p* = .06), and less rainfall the previous day (*p* = .01).

### 3.2 The effects of deer and rodent abundance on the prevalence of Lyme disease pathogens

A total of 7095 questing nymphs were screened for *B. burgdorferi* s.l. (4463 in 2018 and 2632 in 2019) with a mean of 296 nymphs tested per site (ranging from 280 to 301, Table S1). Out of these, 159 nymphs were infected with *B. burgdorferi* s.l. (2.2%, 95% CI: 1.9–2.6, range: 0–9.3%). The predominant genospecies was *B. afzelii* (51.9%, 82/159 of positive nymphs) followed by *B. garinii* (18.2%, 29/159), *B. valaisiana* (10.7%, 17/159) and *B. burgdorferi* s.s. (8.8%, 14/159). Out of these 159 positive samples, 10.7% (17/159) could not be amplified using the nested PCR and thus, could not be sequenced for genospecies identification and were thus excluded from the analysis focusing on *B. afzelii*.

#### 3.2.1 The effects of deer density on the prevalence of *B. burgdorferi* s.l.

The best fit model included deer density the previous year and woodland type: there was a negative correlation between nymphal infection prevalence and deer density the previous year (*p* < .001). For every unit increase in deer density (individual/km^2^), prevalence decreased by 0.06% (Table 2, Figure 4b). Infection prevalence in coniferous woodlands (predicted prevalence: 2.00%, 95%CI: 1.21–3.31) was higher than in deciduous (predicted prevalence: 0.43%, 95%CI: 0.14–1.36; *p* = .03) but similar to mixed woodlands (predicted prevalence: 0.82%, 95%CI: 0.35–1.93; *p* = .10) (Table S4 for Tukey‐tests results).

**TABLE 2:** Outputs from the generalized linear mixed effect model focusing on the effects of deer and environmental factors on nymphal infection prevalence for *B. burgdorferi* s.l.

	Estimate (log)	SE	*z*‐value	*p*‐value	ΔAICc^a^
Intercept	−3.06	0.35	−8.82	<.001	
Deer density year_*t*−1_	−0.06	0.02	−2.90	.004	4.1
Woodland type (baseline: Coniferous)					
Deciduous	−1.55	0.60	−2.59	.009	5.1
Mixed	−0.91	0.44	−2.03	.04	

^a^ The ΔAICc refers to the effect of removing the variable in the given row on the AICc of the best model. For example, a ΔAICc of 10 means that the AICc of the model increased by 10 after removing the variable.

#### 3.2.2 The effects of deer density and rodent abundance on the prevalence of *B. afzelii*

Deer density, rodent abundance, and their interaction were not retained during model selection and therefore, did not affect prevalence of *B. afzelii*. The best model included month only as a predictor (ΔAICc of 1.9 if month is removed).

### 3.3 The effects of host abundance on the density of infected nymphs (disease hazard)

#### 3.3.1 The effects of deer density on the density of infected nymphs with *B. burgdorferi* s.l.

The best model included woodland type and temperature and deer density the previous year was not retained during model selection (Table 3). Lyme disease hazard was higher in coniferous (predicted density: 1.07/100 m^2^, 95%CI: 0.79–1.46) compared to deciduous forests (predicted density: 0.24/100 m^2^, 95%CI: 0.10–0.57; *p* = 0.03) and mixed forests (predicted density: 0.42/100 m^2^, 95%CI: 0.22–0.81; *p* = 0.03) (Table S5). There was a negative correlation between Lyme disease hazard and temperature with the density of infected nymphs decreasing by 0.01 for every 1°C increase in temperature. Lyme disease hazard did not seem to be affected by rainfall the previous day, month, or ground vegetation height.

**TABLE 3:** Outputs from the generalized linear mixed effect model focusing on the effects of deer and environmental factors on the density of nymphs infected with *B. burgdorferi* s.l.

	Estimate (log)	SE	*z*‐value	*p*‐value	ΔAICc^a^
Intercept	−2.43	0.58	4.18	<.001	
Woodland type (baseline: coniferous)					
Deciduous	−1.48	0.45	−2.28	.001	6.67
Mixed	−0.93	0.36	−2.56	.01	
Temperature	−0.13	0.04	−3.64	<.001	8.95

^a^ The ΔAICc refers to the effect of removing the variable in the given row on the AICc of the best model. For example, a ΔAICc of 10 means that the AICc of the model increased by 10 after removing the variable.

#### 3.3.2 The effects of deer and rodent abundance on the density of nymphs infected with *B. afzelii*

The interaction between deer density and rodent abundance and rodent abundance were discarded during model selection and thus, had no effect of the density of infected nymphs with *B. afzelii*. The best model included only deer density the previous year and month as predictors (ΔAICc of 1.5 if deer removed and ΔAICc of 74.2 if month is removed).

## 4 DISCUSSION

Our main aim was to test the effects of deer on Lyme disease hazard over a wide range of deer densities while simultaneously accounting for varying rodent densities. We also wanted to gain mechanistic insight through these hosts' effects on tick density and pathogen prevalence. We found evidence for a dilution effect from deer on infection prevalence for *B. burgdorferi* s.l. and yet, despite this, deer density was not negatively correlated with Lyme disease hazard for *B. burgdorferi* s.l. This was a consequence of a strong positive effect of deer on questing *I. ricinus* nymph density the following year. This demonstrates that a pathogen dilution host can maintain the environmental hazard of disease if it feeds a large proportion of the vector population.

The first mechanism we investigated was the ecological role of hosts in driving vector abundance. As predicted, there was a positive correlation between nymph and deer densities, supporting their well‐documented role as tick reproduction hosts and driving tick population densities (Gandy et al., 2021; Gilbert et al., 2012; Mysterud et al., 2016; Pacilly et al., 2014). We could have expected to observe a quadratic relationship between deer density and nymph density, as described by Kilpatrick et al. (2017) for the North American system. The highest deer density in our study was 31 deer per km^2^, which is below the threshold predicted in that study and could explain why we did not detect this quadratic relationship. However, another study conducted in Scotland investigated the correlation between questing nymphs and deer density and observed a linear correlation, even when deer density reached 50 deer/km^2^, which implies that this threshold might be higher in Europe (Dickinson et al., 2020).

While studies have shown that both red and roe deer can drive tick densities, it would be interesting to separate deer species in future analyses as they might use their habitats in different ways and potentially feed different proportions of each stage of tick and thus have different effects on tick densities, pathogen prevalence and Lyme disease hazard. One study conducted in Sweden found that roe and red deer had similar burdens for larvae and adult females while roe deer harbored more nymphs compared to red deer (Fabri et al., 2021). Ideally, we would have also needed the density of deer 2 years prior to tick collection as adult females would have fed on deer 2 years before the next generation emerged as nymphs, however, it was not logistically possible for this study.

We were expecting nymph density to be positively correlated with rodent abundance the previous year as small mammals can feed up to 89% of larvae in some ecosystems (Hofmeester et al., 2016; Tälleklint & Jaenson, 1994). Interestingly, results suggested that questing nymph density was higher in sites supporting low rodent abundance the previous year. This is unexpected as, when calculating the number of larvae fed (larval burden x rodent abundance using trapping data), we found that sites with high rodent abundance fed almost four times more larvae than sites with low rodent abundance. These results could suggest that other hosts that were not recorded (e.g., squirrels, shrews, birds) might have contributed in feeding larvae the previous year or nymphs the year the survey was conducted. In addition, sites with low rodent abundance generally had high densities of deer and this could suggest that deer can feed a large proportion of immature ticks if no other hosts are available. Furthermore, we only had one site that had high densities of both deer and rodents, probably due to the negative effects that higher densities of deer can have on rodents through direct disturbance and negative impacts on vegetation (Flowerdew & Ellwood, 2001; Gandy et al., 2021). Thus, strong effects of deer on nymph density and, simultaneously, their potential negative effects on rodents, might have masked any effects of rodent abundance on nymph density.

Questing nymphs were also more abundant when the ground vegetation was composed of *Ericaceous* species (such as heathers and *Vaccinium)* and ferns compared to grasses or mosses. These types of vegetation provide mild microclimate for ticks and good cover and food for rodents, who might be more likely to use them, which could explain the higher tick density. Questing nymph density was negatively correlated with vegetation height, which is expected as deep and thick ground vegetation hampers the effectiveness of the blanket drag method (Ruiz‐Fons & Gilbert, 2010). Deciduous woodlands, which can have higher densities of hosts compared to other woodland types (Heyman et al., 2009; Hofmeester et al., 2017), harbored more nymphs compared to mixed forest, in concurrence with previous studies (Estrada‐Peña, 2001; Lindström & Jaenson, 2003; Vourc'h et al., 2016).

Deer may also influence pathogen prevalence in questing nymphs through feeding the larval tick stage the year before we sampled the nymphs. As deer do not transmit *B. burgdorferi* s.l. (Jaenson & Tälleklint, 1992; Kurtenbach et al., 2002), we predicted a negative correlation between deer density and nymphal infection prevalence, and our results supported this, demonstrating that deer in our ecosystem cause a pathogen dilution effect (Rosef et al., 2009; Vourc'h et al., 2016). These findings strengthen the empirical evidence that high deer density can significantly reduce prevalence of *B. burgdorferi* s.l. in some ecosystems which, logically, must be a result of roe and red deer feeding a large proportion of larvae. The strength of this dilution effect of deer on the prevalence of *B. burgdorferi* s.l. should also depend on the abundance of pathogen transmission hosts (Gilbert et al., 2001; Norman et al., 1999). Therefore, we tested the effect of both deer and rodent densities simultaneously on nymphal infection prevalence for *B. afzelii*, which is the genospecies that is transmitted by rodents. While we expected a dilution effect with deer density and a positive effect of rodent abundance on *B. afzelii* prevalence, as a higher proportion of larvae would have fed on transmission hosts when they are abundant, neither variable was retained during model selection. It is possible that contributing factors to this lack of effect could be both from our pathogen data and rodent abundance data. A very low prevalence of *B. afzelii* (1.1%) over our sites greatly reduced the variation needed to detect a statistical effect. In addition, while we estimated the abundance of bank voles and wood mice, there are other mammalian hosts of *B. afzelii* that we did not take into account, such as shrews (Brisson & Dykhuizen, 2004; Mysterud et al., 2019). The relative abundance of rodents is extremely challenging to estimate across contrasting habitats, requiring different methods in different vegetation types. Specifically, live trapping was ineffective in grasses where, instead, it was easy to record vole sign such as tunnels and holes. However, this use of rodent sign may not always reflect current vole activity and this method largely excludes wood mice that do not produce these signs. This highlights the need for improved methods of estimating the relative abundances of rodents and other mammal hosts across a variety of habitats, and the challenges of testing hypotheses about complex host–pathogen ecology. Another study conducted in North West Scotland also found low abundance of rodents in woodlands (mean of 4.67 rodent per 100 trap nights, ranging from 0 to 15.6/100 trap nights), which could highlight a low abundance of wood mice and bank voles in Northern Scotland (Olsthoorn, 2021).

Our results suggested that the prevalence of *B. burgdorferi* s.l. was higher in coniferous woodlands compared to mixed and deciduous forests. While these results might be due to the fact that the majority of our woodlands were coniferous forests (*n* = 17), it could also reflect that species richness might be higher in deciduous woodlands (Sweeney et al., 2010), meaning that the probability of larvae feeding on noncompetent hosts increases (Ostfeld & Keesing, 2000a). In addition, deer might be more abundant in mixed and deciduous woodlands (Hofmeester et al., 2017) and reduce pathogen prevalence through a dilution effect. However, the interaction between deer density and woodland type was not retained during model selection.

The environmental hazard of Lyme disease is a function of the combined effects of hosts on tick density and pathogen prevalence and is therefore also shaped by the abundance of both transmission hosts and nontransmission hosts. We predicted that the lowest Lyme disease hazard should be in ecosystems supporting low densities of both rodents and deer. Notwithstanding insufficient pathogen and rodent data to adequately test the effect of rodents on *B. afzelii*, we found that the density of deer did not have any effects on the density of nymphs infected with *B. burgdorferi* s.l. over our study region. These results show that, even though deer can act as pathogen dilution hosts, significantly lowering nymphal infection prevalence, their positive effects on nymph density resulted in no particular trend of deer density with Lyme disease hazard. This demonstrates the fascinating ecological scenario in which a pathogen dilution host could maintain the environmental hazard of disease when its contribution to vector population density is stronger than its diluting effect on pathogen prevalence.

Although many empirical studies demonstrated a positive association between transmission host abundance and disease hazard (Krawczyk et al., 2020; Ostfeld et al., 2018; Takumi et al., 2019), fewer have investigated the effects of noncompetent hosts. Several studies have reported effects of deer density on Lyme disease hazard or incidence (James et al., 2013; Kilpatrick et al., 2014; Mysterud et al., 2016; Takumi et al., 2019; Vourc'h et al., 2016). However, to our knowledge, few studies designed the surveys and chose sampling sites with the specific aim of testing the effect of a wide variety of deer densities, while also considering rodent abundance. One study in North America suggested that high deer density could lower Lyme disease hazard if the role of deer in dilution pathogen prevalence was stronger than their role in driving tick abundance (Huang et al., 2019), which is what we could have expected in this study. The density of deer in that study was 51–59 deer/km^2^, which is much higher than deer density in our study and could explain why we did not observe such effect. Future studies in Europe should thus, try to include sites with higher deer density if possible. For tick‐borne encephalitis virus (TBEV), two studies explored the effect of the ratio of transmission hosts and noncompetent hosts on disease hazard and found a nonlinear correlation between TBEV and noncompetent host (deer) density as well as an association between deer and TBEV distribution (Bolzoni et al., 2012; Cagnacci et al., 2012).

Our results suggested that Lyme disease hazard was higher in coniferous woodlands compared to mixed and deciduous forests, due to pathogen prevalence being higher. As discussed previously, this could reflect the fact that deer might be using coniferous woodlands less and that deciduous woodlands could have a higher species richness.

While we found that variation in deer density had no effect on Lyme disease hazard, even though they dilute pathogen prevalence, because of their strong effect on tick densities, future resources could be invested in better understanding the highly complex interactions between the many transmission host species and genospecies of *B. burgdorferi* s.l. To further understand Lyme disease ecology and which ecological factors drive other genospecies in the United Kingdom (*B. garinii*, *B. valaisiana* and *B. burgdorferi* s.s.), surveys targeting transmission hosts for these genospecies (e.g., birds, squirrels) are needed. This is a crucial step in understanding how Lyme disease hazard is shaped by the complexities of the ecological interactions between host species, and by the proportions of each tick life stage that feed on each host type.

## AUTHOR CONTRIBUTIONS

**Sara Louise Gandy:** Conceptualization (supporting); data curation (lead); formal analysis (lead); investigation (lead); methodology (equal); project administration (lead); validation (lead); visualization (lead); writing – original draft (lead); writing – review and editing (lead). **Elizabeth Kilbride:** Investigation (supporting); methodology (supporting); resources (equal); writing – review and editing (supporting). **Roman Biek:** Conceptualization (equal); funding acquisition (equal); methodology (equal); project administration (equal); resources (equal); supervision (equal); writing – review and editing (equal). **Caroline Millins:** Conceptualization (equal); methodology (equal); project administration (equal); supervision (equal); writing – review and editing (equal). **Lucy Gilbert:** Conceptualization (equal); funding acquisition (equal); methodology (equal); project administration (equal); supervision (equal); writing – review and editing (equal).

## CONFLICT OF INTERESTS

No conflicts of interests to declare.

## Supporting information


Appendix S1

## Data Availability

Raw data used to produce the results have been deposited on Dryad and are available at: https://doi.org/10.5061/dryad.rv15dv49r.
