# Supplementary material for: No net effect of host density on tick‐borne disease hazard due to opposing roles of vector amplification and pathogen dilution
Source: Ecol Evol. 2022 Sep 6;12(9):e9253. doi: 10.1002/ece3.9253 (PMC9448966; doi:10.1002/ece3.9253)
Supplement: Supplementary file 1 — Appendix S1 [file ECE3-12-e9253-s001.docx]

**Supplementary Information**

Gandy et al., 2022. No net effect of host density on tick-borne disease hazard due to opposing roles of vector amplification and pathogen dilution

Table S1: Table summarising the woodland type, mean deer density, mean rodent index, mean rodent abundance, rodent index used for analysis, mean density of questing nymphs (DON), nymphal infection prevalence for *B. burgdorferi* s.l. (NIP) and the density of infected nymphs (DIN) for *B. burgdorferi* s.l. for each of the 24 sites surveyed.

| # | Habitat | Deer/km² | Rodent index /10m² | Rodent /100TN | Rodent index | DON (/100m²) | NIP % (+ve/tested) | DIN (/100m²) |
| --- | --- | --- | --- | --- | --- | --- | --- | --- |
| 1 | Coniferous | 19.4 | 1.00 | 0 | High | 35.3 | 1.3% (4/301) | 0.65 |
| 2 | Coniferous | 8.5 | 0.75 | 0.50 | Low | 53.0 | 2.3% (7/299) | 1.34 |
| 3 | Coniferous | 19.7 | 0.20 | 0 | Low | 44.2 | 5.7% (17/298) | 2.45 |
| 4 | Coniferous | 15.6 | 5.65 | 4.50 | High | 30.8 | 1% (3/297) | 0.37 |
| 5 | Coniferous | 21.4 | 0.70 | 0.50 | Low | 44.7 | 1.7% (5/300) | 0.83 |
| 6 | Coniferous | 2.0 | 0.20 | 0.87 | Low | 21.8 | 3.4% (10/298) | 0.97 |
| 7 | Deciduous | 8.0 | 0 | 1.23 | Low | 48.5 | 0.7% (2/300) | 0.32 |
| 8 | Coniferous | 15.9 | 0.20 | 1.00 | Low | 52.8 | 4.7% (14/300) | 2.32 |
| 9 | Deciduous | 1.0 | 1.30 | 0 | High | 13.7 | 3% (9/300) | 0.36 |
| 10 | Coniferous | 1.0 | 0 | 1.01 | Low | 16.3 | 9.3% (26/280) | 1.51 |
| 11 | Coniferous | 31.6 | 0.15 | 1.00 | Low | 58.5 | 0.7% (2/298) | 0.44 |
| 12 | Coniferous | 9.0 | 3.65 | 0 | High | 05.0 | 0.7% (2/289) | 0.06 |
| 13 | Coniferous | 10.6 | 0.10 | 3.54 | High | 32.5 | 2% (6/300) | 0.75 |
| 14 | Mixed | 19.8 | 0.50 | 0 | Low | 52.2 | 0% (0/299) | 0.0 |
| 15 | Deciduous | 1.8 | 0.05 | 2.03 | High | 25.8 | 0% (0/291) | 0.0 |
| 16 | Mixed | 19.0 | 0.20 | 1.61 | Low | 32.7 | 1.3% (4/297) | 0.60 |
| 17 | Mixed | 16.4 | 0.20 | 1.36 | Low | 36.3 | 0.7% (2/294) | 0.28 |
| 18 | Coniferous | 4.0 | 0.50 | 0 | Low | 07.7 | 3.2% (9/284) | 0.23 |
| 19 | Coniferous | 15.1 | 0.80 | 9.55 | High | 37.7 | 1.3% (4/300) | 0.42 |
| 20 | Mixed | 15.6 | 0.05 | 5.00 | High | 36.3 | 0.7% (2/299) | 0.25 |
| 21 | Coniferous | 2.0 | 0.75 | 2.75 | High | 11.8 | 5.1% (15/295) | 0.62 |
| 22 | Coniferous | 13.6 | 0.15 | 1.26 | Low | 11.8 | 1.7% (5/293) | 0.21 |
| 23 | Coniferous | 26.5 | 1.15 | 0 | High | 32.7 | 3.5% (10/283) | 0.96 |
| 24 | Coniferous | 30.9 | 0.25 | 0.50 | Low | 112.5 | 0.3% (1/300) | 0.29 |

Figure S1: Distribution and cut-off values to determine rodent indexes (high vs low) for (a) rodent abundance per 100 trap nights and (b) vole index per 10m. Dashed lines represent the threshold values used.

**
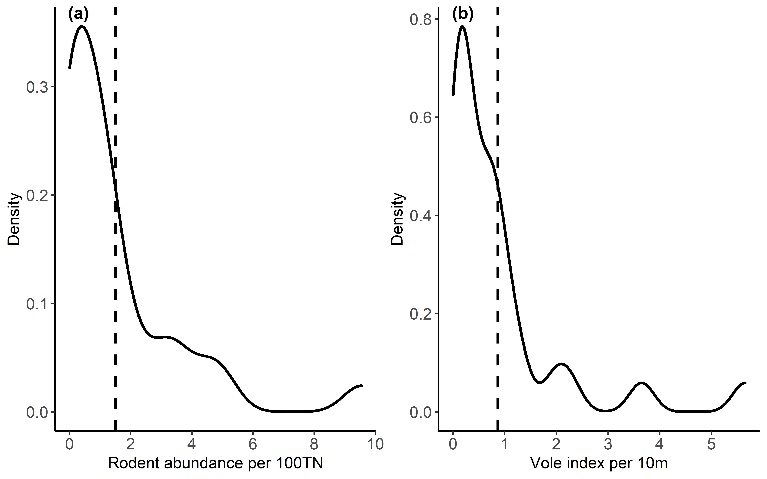
**

Figure S2: Number of larvae fed (relative rodent abundance x larval burden) in sites with high and low rodent abundance.


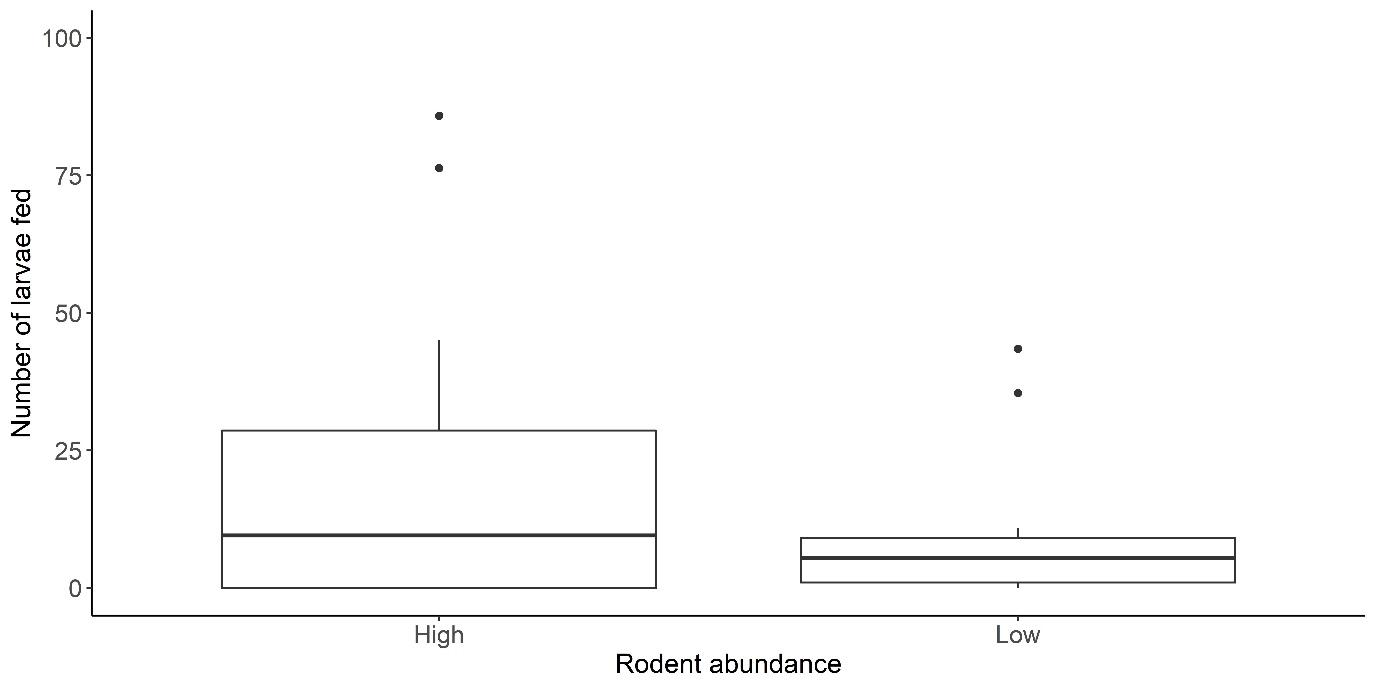


Table S2: Effect sizes (mean prediction and 95%CI) extracted from the output of the GLMM explaining the effects of deer, rodents and environmental factors on the density of questing nymphs.

| Variable | Predicted density of nymph per 100m² [95%CI] |
| --- | --- |
| **Vegetation** |  |
| Bracken | 22.2 [13.7-35.9] |
| *Ericaceous* species | 20.9 [14.1-31.1] |
| Grass | 14.7 [9.9-22.0] |
| Moss | 12.8 [7.8-21.0] |
| **Month** |  |
| July | 15.0 [9.6-23.3] |
| May | 17.2 [11.1-26.8] |
| September | 20.9 [13.5-32.4] |
| **Ground wet?** |  |
| Yes | 26.4 [17.2-40.7] |
| No | 8.9 [5.5-14.3] |
| **Woodland type** |  |
| Coniferous | 24.7 [15.6-39.4] |
| Deciduous | 44.2 [21.7-90.7] |
| Mixed | 16.2 [8.3-32.0] |

Table S3: Results from the Tukey HSD test representing the difference in the average density of questing nymphs (fitted values) depending on ground vegetation type, month and whether the ground was wet during tick collection. For each pairwise comparison, we report the estimate of the difference between the average value of two levels. For e.g. the estimate May – July is positive, so it means the difference in the average number of ticks between May and July is positive and thus, ticks were more abundance in May.

| Comparison | Estimate | Lower 95%CI | Upper 95% CI | p-value |
| --- | --- | --- | --- | --- |
| **Ground vegetation** |  |  |  |  |
| *Ericaceous* species – Bracken | -0.06 | -0.44 | 0.33 | 0.98 |
| Grass – Bracken | -0.41 | -0.78 | -0.04 | 0.02 |
| Moss – Bracken | -0.55 | -1.10 | 0.003 | 0.05 |
| Grass – *Ericaceous* species | -0.35 | -0.56 | -1.14 | <0.001 |
| Moss – *Ericaceous* species | -0.49 | -0.93 | -0.05 | 0.02 |
| Moss – Grass | -0.14 | -0.54 | 0.26 | 0.80 |
| **Woodland type** |  |  |  |  |
| Deciduous – Coniferous | 0.58 | -0.13 | 1.29 | 0.13 |
| Mixed – Coniferous | -0.42 | -1.09 | 0.25 | 0.30 |
| Mixed – Deciduous | -1.00 | -1.90 | -0.10 | 0.03 |
| **Month** |  |  |  |  |
| May – July | 0.13 | -0.03 | 0.31 | 0.14 |
| September – July | 0.33 | 0.17 | 0.50 | <0.001 |
| September – May | 0.20 | 0.02 | 0.37 | 0.02 |
| **Ground wet?** |  |  |  |  |
| Yes – No | -1.09 | -1.31 | -0.87 | <0.001 |
| **Rodent abundance** |  |  |  |  |
| Low – High | 0.49 | 0.11 | 0.87 | 0.01 |

Table S4: Results from the Tukey HSD test representing the difference in the nymphal infection prevalence with *B. burgdorferi* s.l. (fitted values) depending on woodland type. For each pairwise comparison, we report the estimate of the difference between the average value of two levels.

|  | Estimate | Lower 95%CI | Upper 95% CI | p-value |
| --- | --- | --- | --- | --- |
| **Woodland type** |  |  |  |  |
| Deciduous – Coniferous | -1.55 | -2.94 | -0.15 | 0.03 |
| Mixed – Coniferous | -0.91 | -1.95 | 0.13 | 0.10 |
| Mixed – Deciduous | 0.64 | -0.97 | 2.24 | 0.61 |

Table S5: Results from the Tukey HSD test representing the difference in the density of infected nymphs for *B. burgdorferi* s.l. (fitted values) depending on woodland type. For each pairwise comparison, we report the estimate of the difference between the average value of two levels.

|  | Estimate | Lower 95%CI | Upper 95% CI | p-value |
| --- | --- | --- | --- | --- |
| **Woodland type** |  |  |  |  |
| Deciduous – Coniferous | -1.33 | -2.38 | -0.28 | 0.008 |
| Mixed – Coniferous | -0.82 | -1.61 | -0.02 | 0.04 |
| Mixed – Deciduous | 0.51 | -0.72 | 1.75 | 0.59 |
